# Long-Term Effect of Cranial Radiotherapy on Pituitary-Hypothalamus Area in Childhood Acute Lymphoblastic Leukemia Survivors

**DOI:** 10.1007/s11864-016-0426-0

**Published:** 2016-07-30

**Authors:** Cecilia Follin, Eva Marie Erfurth

**Affiliations:** Department of Endocrinology, Skåne University hospital and IKVL, Lund University, Lund, Sweden

**Keywords:** Childhood acute lymphoblastic leukemia, Cranial radiotherapy, Late complications, Pituitary hormone deficiency, Hypothalamic-pituitary axis, Growth hormone, Gonadotropin, Prolactin, Thyroid stimulating hormone, Adrenocorticotrophic hormone

## Abstract

Survival rates of childhood cancer have improved markedly, and today more than 80 % of those diagnosed with a pediatric malignancy will become 5-year survivors. Nevertheless, survivors exposed to cranial radiotherapy (CRT) are at particularly high risk for long-term morbidity, such as endocrine insufficiencies, metabolic complications, and cardiovascular morbidity. Deficiencies of one or more anterior pituitary hormones have been described following therapeutic CRT for primary brain tumors, nasopharyngeal tumors, and following prophylactic CRT for childhood acute lymphoblastic leukemia (ALL). Studies have consistently shown a strong correlation between the total radiation dose and the development of pituitary deficits. Further, age at treatment and also time since treatment has strong implications on pituitary hormone deficiencies. There is evidence that the hypothalamus is more radiosensitive than the pituitary and is damaged by lower doses of CRT. With doses of CRT <50 Gy, the primary site of radiation damage is the hypothalamus and this usually causes isolated GH deficiency (GHD). Higher doses (>50 Gy) may produce direct anterior pituitary damage, which contributes to multiple pituitary deficiencies. The large group of ALL survivors treated with CRT in the 70–80-ties has now reached adulthood, and these survivors were treated mainly with 24 Gy, and the vast majority of these patients suffer from GHD. Further, after long-term follow-up, insufficiencies in prolactin (PRL) and thyroid stimulating hormone (TSH) have also been reported and a proportion of these patients were also adrenocoticotrophic hormone (ACTH) deficient. CRT to the hypothalamus causes neuroendocrine dysfunction, which means that the choice of GH test is crucial for the diagnosis of GHD.

## Introduction

Survival rates of childhood cancer have improved markedly, and today more than 80 % of those diagnosed with a pediatric malignancy will become 5-year survivors [1]. In the 1970s and early 1980s, cranial radiotherapy (CRT) has been part of an effective multimodality therapy to treat as well as prevent the spread of childhood acute lymphoblastic leukemia (ALL). Although radiotherapy was effective, survivors exposed to CRT are at particularly high risk for long-term morbidity, such as endocrine insufficiencies, second malignant neoplasms, metabolic complications, and cardiovascular morbidity [2]. The use of radiotherapy to treat ALL has been reduced to avoid the late occurring complications, but 10–15 % of the contemporary ALL patients will still be exposed [3]. The most commonly diagnosed chronic conditions after CRT involves the endocrine system and in particular the hypothalamic-pituitary-axis [4••, 5•]. Deficiencies of one or more anterior pituitary hormones have been described following therapeutic CRT for primary brain tumors, nasopharyngeal tumors, and following prophylactic CRT for childhood ALL. Studies have consistently shown a strong positive correlation between the total radiation dose and the development of pituitary deficits [6–8]. Further, age at treatment and also time since treatment has strong implications on pituitary hormone deficiencies [9]. It has become apparent that the pituitary hormone deficiencies can develop many years after radiotherapy, and studies have suggested that the damage might be at the level of hypothalamus [10, 11]. There is a difference in the incidence of anterior pituitary hormone deficiencies, with secretion of growth hormone (GH) being the most frequently affected followed by gonadotropin, adrenocorticotrophic hormone (ACTH) and, thyroid stimulating hormone (TSH). Prolactin (PRL) insufficiency is probably an early occurring insufficiency. The most important predictive factors to deficient hormone axes are dose of CRT, age at CRT, and time since CRT.

Hypopituitarism is an important diagnosis to make correctly, and endocrinologists should be involved at an early stage of patient management. It has been shown that untreated pituitary deficiency is associated with poor health. Hypopituitary patients on conventional hormone therapy but without GH therapy have an increased cardiovascular mortality in comparison with the general population [12]. GH is secreted in an intermittent pulsatile pattern from the pituitary and is under control of two interacting hypothalamic factors—one stimulatory, growth hormone releasing hormone (GHRH), and the other inhibitory, somatostatin. GH secretion is regulated by negative feedback by circulating insulin growth factor-I (IGF-I). GH acts on several organs and tissues in the body such as the brain, muscle, kidney, heart, and bone. There are currently consensus guidelines on the diagnostic procedures of GH deficiency published in 2000 by The Growth Hormone Research Society. Luteinizing hormone (LH) and follicle stimulating hormone (FSH), essential for reproduction, are secreted from cells in the pituitary called gonadotrophs and stimulates the gonads in both women and men. The secretion of PRL from the pituitary is inhibited by dopamine and stimulated by thyroid releasing hormone (TRH) from the hypothalamus. TSH secretion is regulated by the interplay of hypothalamic factors TRH and somatostatin and thyroid feedback. The regulator of TSH secretion is feedback by thyroid hormones T_4_ and T_3._ Thyroid hormones act at the hypothalamic level to inhibit TRH synthesis and at the pituitary to inhibit TSH secretion. ACTH secretion by the pituitary is regulated by corticoreleasing hormone from the hypothalamus. ACTH stimulates the adrenal cortex to release cortisol, a life sustaining hormone essential to the maintenance of homeostasis.

The aim of this review is to provide an overview of late hypothalamus-pituitary dysfunction due to cranial radiotherapy in adult survivors of childhood leukemia. We summarize the range of anterior pituitary hormone deficiencies, such as GH, LH, FSH, PRL, ACTH, and TSH, and provide recommendations for diagnosis and surveillance for childhood leukemia survivors.

## Acute lymphoblastic leukemia

Acute lymphoblastic leukemia is the most common type of childhood cancer and is usually treated with chemotherapy such as anthracyclines, vinicristine, asparaginase, and corticosteroids. Further, patients with central nervous system leukemia receive cranial radiotherapy. Importantly, the history of the ALL survivors treated during the 1970s and early 1980s mirrors how to follow these survivors today. Cranial radiotherapy (CRT) was a crucial part of an effective multimodality therapy to treat as well as prevent the spread of childhood acute lymphoblastic leukemia (ALL), which means that everyone diagnosed with ALL during these years received CRT (18–24 Gy) or craniospinal radiotherapy (CSR). Total body irradiation (TBI) has been used since the 1980s in preparation for bone marrow transplantation for relapsed leukemia. Thus, ALL survivors treated with CRT, CSR, or TBI and with any of the following symptoms should be referred for endocrine evaluation: persistent fatigue, obesity, abnormal menstruation, sexual dysfunction, and fragility fractures.

## GH deficiency

CRT is a potent cause of hypopituitarism, and the severity is related to dose, the fraction schedule, and the post-irradiation time interval [11, 13]. CRT will affect the hypothalamus, which is more sensitive to irradiation than is the anterior pituitary [6, 14] and GH deficiency (GHD) is usually the first and often the only established endocrine sequel of CRT [11, 15]. CRT in children frequently causes abnormal hypothalamic-pituitary function later in life [6], and growth deficits have been reported consistently after doses of > 4 Gy CRT [16–18]. However, GHD has also been shown during childhood in ALL patients after low doses of <20 Gy [19, 20], but these data are less consistent [21, 22].

Based on the background to hypothalamic-pituitary disease, different GH tests must be carefully considered [23]. There are clear cut off levels for GH when a GHD is diagnosed, i.e., for the insulin tolerance test (ITT) the level is GH <3 ug/l (or 9 mU/L), and for the growth-hormone releasing hormone (GHRH)-arginine test which is BMI dependent, we used the same cut off levels [24]. Thus, in accordance with Darzy et al. [23], Björk et al. [24] also recorded that the ITT clearly reflected the presence of early radiation-induced GHD, but this was not always the case with the GHRH-arginine test, which more confirmed the diagnosis later in life. The GHRH-arginine test is more a stimulation test directly on the pituitary, reflecting the pituitary GH secretion. However, when the GH response to GHRH-arginine was low, we considered the patient to be clearly GHD [24]. Thus, it would appear that primarily the hypothalamus and then later direct pituitary damage from CRT was the cause of GHD among the former ALL patients.

**Fig. 1:**
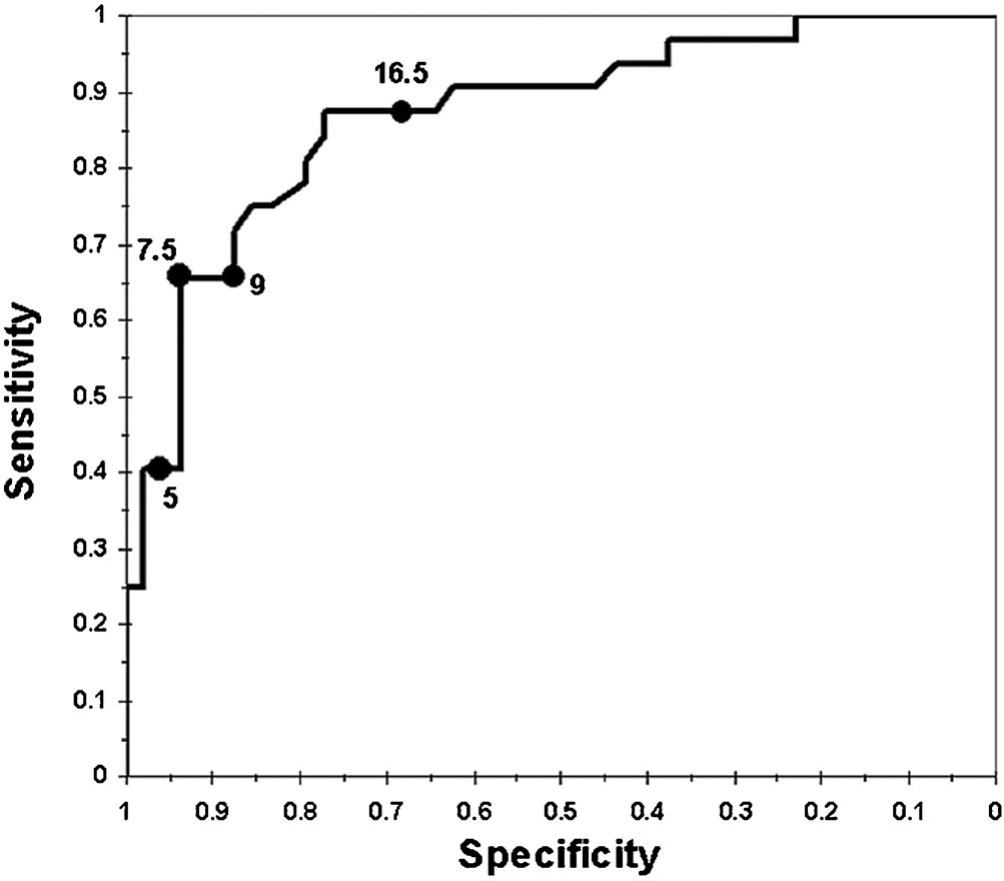
Receiver operating characteristic curve of the GHRH-arginine test for classifying severe GHD, defined as ITT below 3 μg/L. The combination of sensitivity and specificity of the peak GH cut off level of 9 μg/L is marked with *filled circle*. Note that the specificity-axis is labeled in descending order.

Brennan et al. [20] investigated GH secretion after CRT in 32 adults, 6.8–28.6 (median 17.8 years) years since CRT. Nine of the patients were severely GHD (peak GH response <9 mU/l to both provocative agents arginine or ITT), and a further 12 patients were GH insufficient (peak GH response <20 mU/l to both tests with at least one peak GH response >9 mU/l). They had all received between 18–24 Gy. A group of 44 childhood ALL with a median of 25 years [19–32] of whom all were treated with CRT median 24 Gy (range 18–24 Gy) were investigated. They were treated with CRT at a median age of 5 years (range 1–18), and 17 years had passed since ALL treatment and CRT [9]. According to the ITT and or the GHRH-arginine test, 91 % were considered GHD. All patients with a peak GH 3.9 μg or more on the GHRH-arginine test performed an ITT (Fig. 1).

Among 310 childhood cancer survivors, of whom 34.5 % were former ALL survivors, the most frequent hormone deficiency was gonadal, primary hypothyroidism and GHD [5•]. GHD was evaluated using standard GH tests. GHD was found among 16.13 %, where of the most frequent background diagnosis was brain tumors, followed by hematological malignancies.

In a recent paper from Chemaitilly et al. [4••] on a mixed population of adults with childhood cancer, with 72 % leukemia diagnosis, about 46.5 % were diagnosed with GHD. However, GHD was defined only as a measurement of morning serum IGF-I <−2.0 *z*-score. This is probably a strong underestimation as serum IGF-I is not considered sufficient to set the diagnosis of GHD [25–27].

## Gonadotropin deficiency

Gonadotropin deficiency in childhood cancer survivors is most frequent after a radiation dose to the hypothalamus-pituitary axis of >40 Gy [28, 29] and can be presented with a spectrum from subclinical to severe impairment. It is the second most common anterior pituitary hormone deficiency after CRT. Clinically significant gonadotropin deficiency after CRT is often apparent after long-term follow-up with an incidence of 20–50 % [6, 30, 31].

Chemaitilly et al. [4••] investigated a mixed population of adults treated for childhood cancer of which 72 % were ALL survivors. LH and FSH deficiency was recognized in 10.8 % of the total cohort of survivors 27 years after cancer diagnosis. The definition of LH/FSH deficiency in men was a testosterone less than 200 ng/dL (10 nmol/L) including a LH less than 7 IU/L and FSH less than 9.2 IU/L. For the women, the definition was amenorrhea in women less than 40 years old with an estradiol less than 17 pg/ml and FSH less than 11.2 IU/L. After CRT doses more than 40 Gy, the prevalence of LH and FSH deficiency was 22.7 and 7.8 % after CRT doses of less than 40 Gy [4••]. Male sex, CRT dose >22 Gy and BMI >30 kg/m were associated with higher odds of LH and FSH deficiency. Results from a recent study, only including men, of 199 childhood cancer survivors showed that 13 survivors had central hypogonadism 14 years after cancer diagnosis. Survivors with testosterone less than 3 ng/dL were considered to have hypogonadism [5]. The risk of hypogonadism was higher in survivors treated with CRT. ALL survivors treated with a moderate dose of CRT of 18–24 Gy have an increased risk of precocious puberty [31, 32]. On the other hand, a large study of 949 female ALL survivors found craniospinal radiotherapy to be associated with an increased risk of late onset menarche [33]. Thus, both early and delayed puberty can be seen.

## PRL deficiency

Hyperprolactinemia after treatment with CRT for childhood cancer has been reported in studies investigating children treated with a dose >40 Gy after a short follow-up [6, 34]. The hyperprolactinemia is due to the reduction in the neurotransmitter dopamine which has an inhibitory effect on PRL secretion. On the other hand, hypoprolactinemia has also been recorded in ALL survivors after CRT, but after long-term follow-up [35]. It has been suggested that severe PRL deficiency occurs late after all other anterior pituitary insufficiencies in the evolution of hypopituitarism and that very low levels of PRL are related to the severity of hypopituitarism [36]. Littley et al. [11] showed that after CRT to adults basal PRL showed an early rise, followed by a gradual decline after a few years. CRT seems to cause a primarily diminished inhibition of PRL secretion resulting in increased basal PRL levels, followed by a slowly developing lactotroph dysfunction. In contrast, in rats exposed to non-fractionated CRT, GH and PRL were shown to be most sensitive of all pituitary hormones, with a dramatic decrease with time and dose after irradiation [37]. It has been shown that PRL deficiency, thus very low PRL levels, was independently associated with reduced levels of serum IGF-I in severely GHD adults and that PRL deficiency can act as a surrogate marker for the severity of GHD [36]. Dunkel et al. found normal basal but subnormal PRL response after metoclopramide in ALL children treated with CRT [38]. Follin et al. recorded significantly lower basal PRL levels and PRL area under the curve (AUC) after GHRH-arginine stimulation test in 44 ALL survivors compared to matched controls of both gender [35]. Seven ALL women reported pregnancies during follow-up, and six out of seven women reported failure to lactate. Thus, ALL patients treated with CRT were not only GH deficient but also PRL insufficient 20 years [8–27] after diagnosis [35].

## TSH deficiency

There are conflicting results in early studies, with no risk [39] or an increased risk [40, 41] of central hypothyroidism after CRT in ALL children treated with <30 Gy. Some previous studies have, however, limitations with a short follow-up period, of 6–9 years [36, 37] and the use of self-reported thyroid function [36]. After a follow-up of an average of 6 years after ALL treatment, one out of the 33 children was found to have a papillary carcinoma of the thyroid. Thyroid function was normal in all patients, with the exception of one case which showed high basal levels TSH (9.2 microU/mL) levels, but normal response to TRH (TSH = 17.8 microU/mL). This hormonal alteration was later normalized [40]. In a study with 8 years of follow-up of survivors of childhood ALL treated with prophylactic CRT, no adverse effect on hypothalamo-pituitary-thyroid function was recorded [41].

Hypothalamic-pituitary-thyroid dysregulation after CRT, identified by a TRH-stimulation test or TSH surge, has been recognized in as many as 15 % of former ALL patients 10 years after CRT [33]. In a recent study from Follin et al. [35] with a median of 20 years of follow-up, no significant difference in basal TSH and only slight disparity of free T_4_ levels (higher), which indicates no known clinical significance, was shown. However, a slightly lower TSH response to a TRH test, shown in 13 ALL patients, might be an early indication of TSH dysfunction. This is in contrast to Darzy et al. [23] who, 11.5 years after cancer treatment (with mixed diagnosis), found increased TRH-stimulated TSH levels in cranially irradiated patients with GHD as compared to matched controls. Disparity in results may be due to differences in radiation dose, type of cancer (34 patients treated for brain tumors and 3 for ALL), age at cancer diagnosis, and particularly in follow-up-time. GH, and thus GHD, plays a role in the regulation of thyroid hormone metabolism, with both a central effect, with an increase in somatostatin inhibition of TSH secretion [42], and a peripheral effect with an increased conversion of free T_4_ to free T3.

In another study of 310 childhood cancer survivors (CCS), of whom 34.5 % were former ALL survivors, the most frequent hormone deficiency was gonadal, primary hypothyroidism, and GHD, after a median time of 16 years since diagnosis [5•]. They documented at least one endocrine disease among 50 % of CCS. Primary hypothyroidism was diagnosed among 17.7 %, whereas seven patients had central hypothyroidism.

Chemaitilly et al. [4••] investigated a mixed population of adults with childhood cancer with a mean of 27 years (10–48) after treatment, with 72 % leukemia patients. TSH deficiency was defined as <4.0 mIU/L and a FT4 <0.9 ng/dL (11 nmol/L). No TRH test was performed. With these baseline results, 7.5 % were considered TSH deficient [4••].

## ACTH deficiency

Symptoms of ACTH deficiency is often subtle such as poor weight gain, anorexia, and fatigue and should be separate from primary adrenal insufficiency presenting with electrolyte imbalance, vitiligo, and hyperpigmentation. Clinically apparent ACTH deficiency is uncommon after CRT. After a total radiation dose of <50 Gy to the H-P axis, around 3 % of childhood brain tumor survivors present with ACTH deficiency short time after (3–10 years) after diagnosis [6, 34]. After radiation doses of >50 Gy the frequency of ACTH deficiency is significantly increased in survivors with head and neck tumors with rates of 27–35 % up to 15 years after treatment [13, 43]. In a study including 310 CCS with mainly hematological malignancies, the authors found four survivors with central hypoadrenalism. Among the 310 survivors, 74 were treated with CRT in childhood. The survivors were screened with morning cortisol, and ACTH and if they were presented with abnormal levels they were tested with proper stimulation tests [5•]. Chemaitilly et al. (year) investigated a mixed population of 748 CCS 27 years after CRT and of which 72 % were ALL survivors. The definition of ACTH deficiency was a cortisol <5 μg/dl (135 nmol/L), and the prevalence of ACTH deficiency in this population was 3.9 % [4••].

In a homogenous group of ALL survivors, treated with a moderate dose of CRT (18–24 Gy), Follin et al. report ACTH insufficiency [44]. Fourteen out of 37 (38 %) ALL survivors had a subnormal cortisol response to an insulin tolerance test (ITT) (257–478 nmol/L; normal >500 nmol/L) while there was no significant difference in basal cortisol levels between 44 ALL survivors and healthy matched control subjects. ALL women, but not ALL men, had significantly lower ACTH levels compared to gender matched controls. However, only a few survivors needed regular hydrocortisone replacement.

## Conclusion

CRT is a potent cause of hypopituitarism, and the severity is related to dose, the fraction schedule, and the post-irradiation time interval. Childhood ALL survivors treated with CRT often develop dysfunction of the hypothalamic-pituitary axis with a spectrum of hormone deficiencies that develop over several years. As GH is the most sensitive hormone to CRT, followed by gonadal, PRL, adrenal, and thyroid, the vast majority of ALL survivors suffer from GHD. The hypothalamus is more sensitive to CRT than the pituitary and causes neuroendocrine dysfunction, which means that the choice of GH test is crucial for the diagnosis of GHD. Further, after long-term follow-up, insufficiencies in PRL and TSH and also ACTH have been reported. Life-long surveillance of the entire hypothalamic-pituitary axis is recommended in ALL survivors treated with CRT.
